# Systematic Exploration of Natural and Synthetic Flavonoids for the Inhibition of *Staphylococcus aureus* Biofilms

**DOI:** 10.3390/ijms141019434

**Published:** 2013-09-25

**Authors:** Suvi Manner, Malena Skogman, Darla Goeres, Pia Vuorela, Adyary Fallarero

**Affiliations:** 1 Pharmaceutical Sciences, Department of Biosciences, Abo Akademi University, Artillerigatan 6A, 3rd Floor, Biocity, Turku FI-20520, Finland; E-Mails: smanner@abo.fi (S.M.); malena.skogman@abo.fi (M.S.); pia.vuorela@abo.fi (P.V.); 2 Center for Biofilm Engineering, Montana State University, Bozeman, MT 59717, USA; E-Mail: darla_g@erc.montana.edu; 3 Division of Pharmaceutical Biology, Faculty of Pharmacy, University of Helsinki, Viikinkaari 5E, P.O. Box 56, Helsinki FI-00014, Finland

**Keywords:** flavonoids, flavanones, flavans, *Staphylococcus aureus*, biofilms, antibacterial, bioactivity, chalcones, flavones

## Abstract

When single-cell (or suspended) bacteria switch into the biofilm lifestyle, they become less susceptible to antimicrobials, imposing the need for anti-biofilms research. Flavonoids are among the most extensively studied natural compounds with an unprecedented amount of bioactivity claims. Most studies focus on the antibacterial effects against suspended cells; fewer reports have researched their anti-biofilm properties. Here, a high throughput phenotypic platform was utilized to screen for the inhibitory activity of 500 flavonoids, including natural and synthetic derivatives, against *Staphylococcus aureus* biofilms. Since discrepancies among results from earlier antibacterial studies on flavonoids had been noted, the current study aimed to minimize sources of variations. After the first screen, flavonoids were classified as inactive (**443**), moderately active (**47**) or highly active (**10**). Further, exclusion criteria combining bioactivity and selectivity identified two synthetic flavans as the most promising. The body of data reported here serves three main purposes. First, it offers an improved methodological workflow for anti-biofilm screens of chemical libraries taking into account the (many times ignored) connections between anti-biofilm and antibacterial properties. This is particularly relevant for the study of flavonoids and other natural products. Second, it provides a large and freely available anti-biofilm bioactivity dataset that expands the knowledge on flavonoids and paves the way for future structure-activity relationship studies and structural optimizations. Finally, it identifies two new flavans that can successfully act on biofilms, as well as on suspended bacteria and represent more feasible antibacterial candidates.

## 1. Introduction

One of the most ground-breaking advancements of the microbiological research during the past 40 years has been the recognition of bacterial biofilms as the predominant bacterial lifestyle instead of bacterial suspensions [1,2]. Bacterial biofilms is the term used to describe the surface-attached bacterial lifestyle. Cells in biofilms grow as communities, surrounded by a self-produced thick layer of extracellular polymeric substances (EPS, also known as matrix or slime). Biofilms are structurally and also functionally different from single-cell (suspended) bacteria [3]. The presence of the EPS protects cells in biofilms from the detrimental effects of chemical insults and harsh environmental conditions. Moreover, in the complex tri-dimensional architecture of biofilms, subpopulations of cells co-exist in all stages of growth, including a fraction of dormant cells that are not metabolically active. This combination of factors helps to explain why biofilms possess much lower susceptibility to antimicrobial therapy or biocides, when compared to suspended cells [4–6]. Biofilms are involved in a wide range of infections, such as chronic wounds [7,8], otitis [9], cystic fibrosis [10] and those associated with medical devices [11], and they are claimed to be responsible for an overwhelming proportion of persistent, antibiotic-resistant infections.

Current challenges faced within the anti-biofilms field are enormous and are present at different stages in the drug discovery process. From a pre-clinical perspective, a limited repertoire of molecules has been reported that can act *in vitro* on existing biofilms at low concentrations, especially in the case of those formed by *Staphylococcus aureus* [12]. Then, even if some compounds can successfully enter the drug discovery pipeline, another more serious challenge is the absence of a clearly-defined regulatory pathway for those products to be registered with relevant agencies, such as the European Medicines Agency, the US Food and Drug Administration or the US Environmental Protection Agency. As a result, only one disinfectant (commercially sold by Sterilex, a US company) and no antibiotics have been approved by a regulatory agency to be used specifically against bacterial biofilms. Clearly, expanding the search for anti-biofilms, as well as consistently documenting their benefits with convincing results is deemed as an imperative need of current biomedical research in the hopes of delivering tangible solutions to biofilm infections.

Flavonoids are one of the most widely recognized groups of natural products, even outside the scientific community. They are largely represented in the human diet, as they are present in plants, seeds and various foodstuffs. From a chemical viewpoint, flavonoids are phenolic compounds that consist of two benzene rings (A and B) combined with an oxygen-containing heterocyclic benzopyran ring (C). Flavonoids can be divided into different classes based on their molecular structure. The number of these classes varies according to the classification criteria. For instance, according to the position of the phenyl ring (B) relative to the benzopyran moiety, they can be classified as flavonoids (2-phenyl-benzopyrans), isoflavonoids (3-phenyl-benzopyrans) and neoflavonoids (4-phenyl-benzopyrans) [13]. Oxidation and saturation status in the heterocyclic ring also enables division of flavonoids into flavans, flavanones, dihydroflavonols, flavonols, flavones, flavone-3-ols and flavone-3,4-diols, while, depending upon the type, number and arrangement of substituents, flavonoids can be further divided into other groups, such as anthocyanidins and chalcones [14]. So far, more than 6500 flavonoids have been discovered, and a total of 14 classes have been proposed by Cushnie and Lamb [15]. From a biological perspective, flavonoids are one of the most extensively studied types of natural compounds, with a massive amount of research published supporting an unprecedented amount of bioactivity claims. In the field of anti-infectives, flavonoids have been reported to display antibacterial, antiviral, antiprotozoan and antifungal properties [16,17]. Most of the studies performed against bacterial infections have focused on suspended cells, with flavones, chalcones, flavonols, flavan-3-ols, flavanones and flavolans as the most successful flavonoid classes [15,18,19].

**Figure 1:**
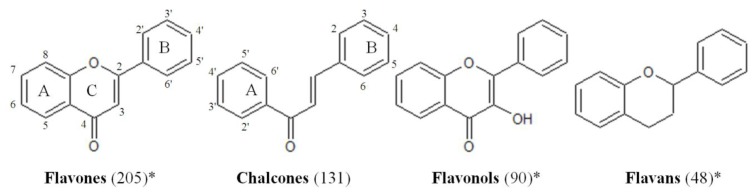
The most important flavonoid classes represented in the flavonoids chemical library. The amount of compounds per class present in the library is indicated between parentheses. Flavanones, isoflavonoids, neoflavonoids and dihydroflavonols are also included. * The numbering of the chemical structure of the flavonols and flavans is similar to the flavones, and it follows the criteria of [15].

This article focuses on the use of flavonoids as a representative chemical template to perform a large screening in search for compounds that can counteract *S. aureus* biofilms. The screened collection consisted of a commercial set of 500 natural and synthetic flavonoids that covers various structural classes (Figure 1) and includes many well-known dietary flavonoids. To the best of our knowledge, the current contribution represents the largest systematic screening study performed with flavonoids for anti-biofilm effects. Compounds per class present in the library is indicated between parentheses. Flavanones, isoflavonoids, neoflavonoids and dihydroflavonols are also included.

## 2. Results and Discussion

### 2.1. Design of the Flavonoid Anti-Biofilm Screening for More Meaningful Data Generation

Earlier studies dealing with the antibacterial properties of flavonoids performed by different groups have generated conflicting data. However, a seminal contribution by Cushnie and Lamb in 2005 [18] elucidated the major reasons that could explain such contradictions, namely, differences in the types of assays used, a lack of optimization or even a failure to enumerate the starting bacterial concentrations, differences in the solvents used to dissolve the flavonoids, as well as in the origin of the compounds (*i.e*., if they are obtained from a commercial or a natural source). Taking this into account, one of the aims of this study was to design chemical screening to minimize these sources of variation.

Here, the strategy that was adopted for anti-biofilm screening against *Staphylococcus aureus* spp. includes a parallel exposure scheme in which bacteria are treated with the compounds before biofilm formation takes place (prior-to-exposure) and once biofilms are formed (post-exposure paradigm). This strategy has been described in the earlier contributions of our group [20–23], and a detailed scheme is presented in [22]. It solely relies on optimized methods to quantify biofilm viability (with resazurin staining) and biomass (with crystal violet staining) under repeatable conditions that have been summarized here and are also detailed in earlier contributions [20–23]. Key conditions, such as biofilm formation time, starting bacterial concentrations or available surface for biofilm formation, are comparable to those that have been applied in anti-biofilm studies on 96-microwell plates [24,25], making inter-laboratory comparisons feasible. Special attention was paid to control the initial concentration of added bacteria, as it is one of the most critical factors in antimicrobial assays [25], as well as to maintain it throughout the screening and follow-up studies. Unlike the antibacterial assays against planktonic bacteria for which straightforward guidelines exist from the Clinical and Laboratory Standards Institute, as well as from the European Committee on Antimicrobial Susceptibility Testing (EUROCAST), only one standard for an anti-biofilm screening assay in microwell plates has been accepted so far by the American Society for Testing of Materials (ASTM E2799-12), and it does not apply to *Staphylococcus aureus* biofilms.

To avoid identifying only strain-specific hits, the initial screening was performed simultaneously against two clinical *S. aureus* strains, and only those flavonoids highly active against both strains and in both exposure paradigms (prior-to and post-biofilm formation) were chosen for further study (selection criteria are discussed in Section 2.2). Moreover, all the tested compounds were obtained from the same company (TimTec, see Section 3.1), to avoid differences due to the source. In Table S1, a list of the flavonoids library is included. Their structures can be retrieved with the company codes using the freely available TimTec database (http://www.echemstore.com/). Trivial names of the natural flavonoids, as well as short names of the natural derivatives were also compiled and included in this Table. All the 500 flavonoids used in this study were prepared in 20 mM in dimethylsulfoxide (DMSO), as this is the most widely used solvent for the solubilization of large chemical libraries. Precipitation upon dilution in DMSO was recorded for 31 flavonoids (Table S1). All the precipitated samples corresponded to being inactive, most likely because precipitation lowers the compound concentration in the solution, resulting in an underestimation of the anti-biofilm activity [26]. However, the population of non-entirely solubilized flavonoids accounted for less than 6.5% of the total collection and, therefore, has a minimal impact on the usefulness of this study.

### 2.2. Anti-Biofilm Screening: Inactive and Moderately Active Flavonoids

**Figure 2:**
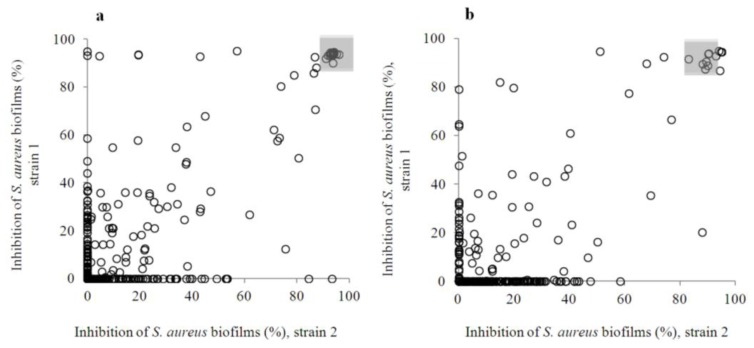
Anti-biofilm effects of the flavonoids collection when added prior-to (**a**) or post-biofilm formation (**b**). Strains 1 and 2 are *S. aureus* ATCC 25923 and Newman clinical strains, respectively. Highly active flavonoids are those present in both shadowed areas. Primary screening results are presented in Table S1.

**Scheme 1:**
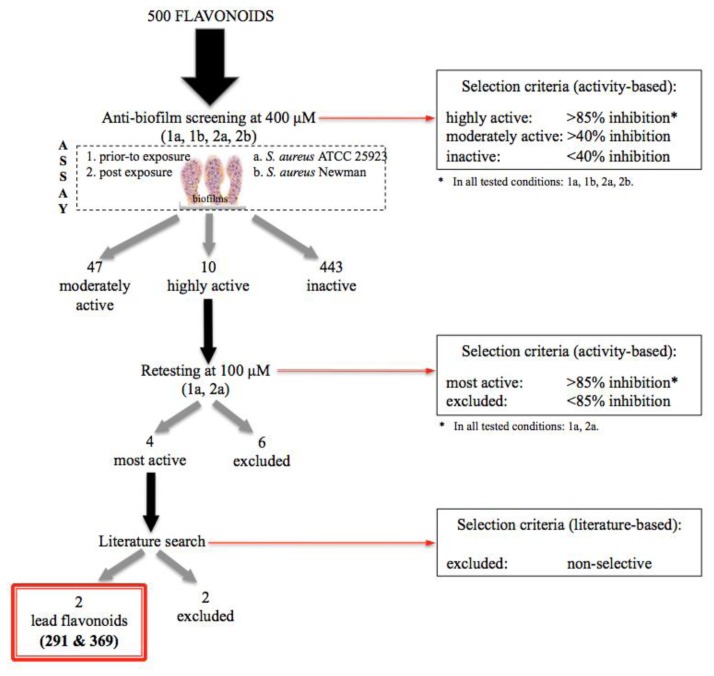
Summary of the anti-biofilm screening and selection criteria applied to the collection in this study. Highly and moderately active flavonoids are listed in Tables S2 and S3, respectively.

Results of the inhibitory activity of the entire flavonoids collection prior-to (a) and post-(b) biofilm formation based on viability results are shown in Figure 2. The selection process is summarized in Scheme 1, and the raw screening data are presented in Table S1.

More than 80% of flavonoids (**443**) caused less than 40% inhibition of the biofilm formation by *S. aureus* (Figure 2) at 400 μM, and they were declared inactive. In general, many flavonoids had strain-specific effects, and their inhibition percentages were lower in the post-exposure paradigm. Upon closer inspection of the screening results (Table S1), ten flavonoids were tentatively identified as highly active, causing more than an 85% inhibition of biofilm viability (based on resazurin staining), when measured using both exposure assays, as well as both *S. aureus* strains. This corresponds to the compounds that are present in both of the shadowed areas marked in the Figure 2, and they are compiled in Table S2. The calculated overall hit rate, based on the amount of compounds identified as highly active, was 2%.

Between the inactive and highly active flavonoids, **47** were found to inhibit biofilm viability more than 40%, and they were classified as moderately active. Flavonoids that displayed activity in only one assay condition (for instance, compound **231**) were intentionally excluded. Thus, all the flavonoids within the moderately active group also displayed varying levels of activity in the other assay conditions. Eight of the moderately active flavonoids were only active in the pre-exposure scheme, and five showed strain-selectivity. A list of the moderately active ones is presented in Table S3.

Observations were first made on the group of inactive flavonoids (Table S1). Dietary flavonoids, present in citrus, such as kaempferol (**69**) and naringenin (**164**), have been proven to act as quorum sensing (QS) inhibitors by interfering with the interaction between acyl-homoserine lactones (AHLs, the signal molecules of Gram-negative bacteria) and their receptors, leading to inhibition of biofilm formation by *Escherichia coli* O157:H7 and *Vibrio harveyi* BB120 [15,27], but they were classified as inactive in this study. Other QS inhibitors, such as baicalein (**159**) and catechin (**160**), were found inactive, as well, when, previously, they had been shown to act on the cytoplasmic membrane-associated receptors, TraR and RhlR, respectively, in this way suppressing biofilm formation by *Pseudomonas aeruginosa* [28,29]. The lack of activity of all these compounds in *S. aureus* biofilms would thus suggest that they may preferentially act in Gram-negative bacteria.

Several flavonoids present in the moderately active group have been extensively studied and were earlier reported with different bioactivities in a variety of targets, including antimicrobial activity (Table S3). However, a total of 20 flavonoids in this group had not been reported as active in the PubChem Compound (http://www.ncbi.nlm.nih.gov/pccompound), the largest publicly available repository for small molecular weight molecules, which is connected to their bioactivities via the PubChem Bioassay project (http://www.ncbi.nlm.nih.gov/pcassay). Thus, 20 new bioactive flavonoids were identified as a result of this contribution.

Chalcones and flavones are the most highly represented flavonoid classes (Table S2) with 12 and 14 moderately active ones, respectively. This could be a reflection of the fact that these two chemical classes were also the most abundant within the collection (Figure 1). Within the chalcones, isoliquiritigenin (**372**) has been earlier shown to possess potent antibacterial properties against suspended *Ralstonia solanacearum* [30] and biofilm-forming *Porphyromonas gingivalis* [31], both Gram-negative bacteria, but no information had been published concerning the effects against Gram-positive bacteria. Chalcones typically exhibited activity prior to biofilm formation, which was not surprising, as many of them had been reported to exhibit antibacterial properties. Eight of them (**189**, **267**, **372**, **466**, **467**, **470**, **474**, **477**) are hydroxylated in position 2′ in the A ring, which has earlier been shown to benefit their antibacterial effects [32,33]. Four share a hydroxylation in position 4′ in the A ring (**189**, **372**, **470** and **477**), and this hydroxylation is also present in three other chalcones (**251**, **425** and **475**); thus, within the moderately active chalcones, a total of 11 compounds (out of 12) have hydroxylations in positions 2′ or 4′ in the A ring. In position 4 of the B ring, hydroxylation (**189**, **267** and **372**) or methoxylation (**251**, **425** and **477**) seemed to be also a relevant structural feature, but it always occurred in conjunction with the substitutions in the A ring discussed earlier. Thus, this may be important, but not sufficient.

**Figure 3:**
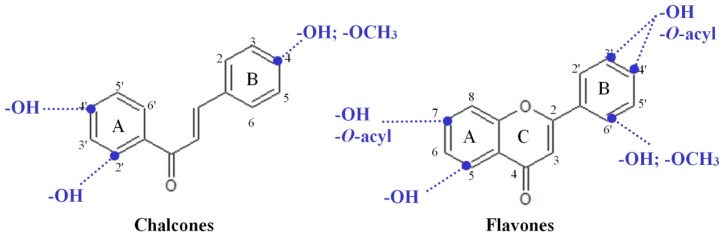
Key structural features present in the anti-biofilm chalcones and flavones (moderately and highly active ones).

In the case of flavones, substitutions in the A ring in positions 7 (-*O*-acyl or -*O*-alkylamino) and 5 (–OH) had been indicated as crucial for antibacterial activity [15,34], and they are indeed present in compounds **52** and **54** (7, -*O*-acyl) and in **56** and **311** (5, –OH). However, it was more commonly present in the hydroxylation of position 7 in the A ring, which occurs in **56**, **121**, **135**, **166**, **188**, **222**, **232** and **311** (eight out of 14 flavones). In two (**166** and **232**), only hydroxylations in the A ring are present (no substituents in the B ring), and one of them (**166**, 7,8-dihydroxyflavone) was found active in the two exposure conditions and in both *S. aureus* strains, with inhibition percentages ranging from 61.5% to 93.3%. The impact of substitutions in the B ring of flavones has been much less studied in the literature. Here, it was found that hydroxylation or *O*-acylation in positions 3′ (**52**, **54**, **56**, **211**, **214** and **222**) or 4′ (**52**, **54**, **56**, **211**, **214** and **311**) were beneficial. The essentiality of the B ring substitution is supported by flavone 214 (3′,4′-dihydroxyflavone), which displayed high inhibition values of biofilm formation by *S. aureus* 25923 strain (over 64%) in the absence of any other changes in the A ring. Additionally, four moderately active flavones also share a substituent (–OH or –OCH_3_) in 6′ (**110**, **121**, **129** and **135**). These indicated motifs in the B ring could be valuable guidance for future chemical refinements on the flavones scaffold. The key structural features for chalcones and flavones, which have been discussed above, are summarized in Figure 3.

Moreover, seven moderately active flavonols (the third most represented flavonoid class after flavones and chalcones) were identified within the moderately active flavonoids. Simultaneous hydroxylations of ring A and B in various positions occurred in all flavonols, except in one (**155**), where only –OH groups were present in the A ring. Among these flavonols, quercetin (**58**) was identified. Quercetin is one of the most extensively studied dietary flavonoids and natural compounds in general. One interesting aspect is that quercetin was found slightly more effective in pre-existing biofilms. Quercetin has been reported to be antibacterial and anti-biofilm against *S. aureus*. In fact, Lee *et al*. [35] recently showed that it inhibits biofilm formation (prior-to exposure) by *S. aureus* strains, including ATCC 25923, at concentrations within the range of 17–170 μM. Thus, the activity found here is lower than the one reported earlier [35]. Unfortunately, [35] included no information on the solvent used to dissolve the quercetin, and it has been shown that quercetin is highly prone to aggregation, depending upon the vehicle, ionic strength and pH [36]. Formation of aggregates has been postulated as one possible reason for quercetin’s promiscuous activity in a variety of assays [37] and may be one explanation for the discrepancy between the results of [35] and the current study. Similarly, the pentaacetate derivative of quercetin (**54**) was identified here to be moderately active (Table S1), but it was on the boundary of being classified as highly active, causing more than an 80% inhibition of biofilm formation in all the assay conditions. Lastly, fisetin (**162**) was found as a moderately active flavonol and had been earlier reported to specifically inhibit *S. aureus* 8324 biofilms at concentrations within the range of 28–56 μM with negligible effects on planktonic bacteria [38].

Novel information was also gathered for other natural flavonoids. The flavonol, galangin (**155**), and the isoflavone, osajin (**288**), were also highly effective in the pre-exposure assay and inhibited the viability of existing biofilms. In the first case, galangin (**155**) has been previously reported as a potent antibacterial against suspended *S. aureus* [26], which could reasonably explain its effectiveness in the pre-exposure assay. No prior reports of the antibacterial properties of osajin (**288**) have been found, but claims of its antioxidant activity have been supported [39].

### 2.3. Highly Active Flavonoids and Further Selection Process

The ten compounds that were identified as highly active, according to the initial screening results, are listed in Table 1. Their structures and bioactivities, as described in the PubChem Compound database are compiled in Table S2.

Six natural flavonoids are present in the highly active group (**59**, **139**, **424**, **446**, **464** and **469**). The first two (**59**, **139**) are discussed further in this chapter. Compound **446**, isosakuranetin, ((2*S*)-5,7-dihydroxy-2-(4-methoxyphenyl)-2,3-dihydrochromen-4-one)), has been isolated from the flowers of *Chromolaena odorata*, and it has been reported to possess moderate anti-mycobacterial activity with minimal inhibitory concentrations (MIC) of 174.8 μM [40]. On the other hand, compound **464** (2′,4′-dihydroxychalcone) has been recently demonstrated as the most active constituent of the plant, *Muntingia calabura*, against suspended *S. aureus* (the same strain used in the current study) with an estimated MIC value of 50 μg/mL (208 μM) [41]. Thus, at 400 μM, the antibacterial activity of **464** on suspended cells could be one possible mechanism for the prevention of biofilm formation registered in the current study. None of these highly active flavonoids have been previously reported to possess anti-biofilm properties (Table S2). The chalcones in this group (**424**, **464** and **469**) have hydroxylation in positions 2′ and 4′, as previously noted in the group of moderately active ones. Because these chalcones were also effective in existing biofilms, albeit only at 400 μM, it could be that these features play a role not only for antibacterial, but also for anti-biofilm activity.

**Figure 4:**
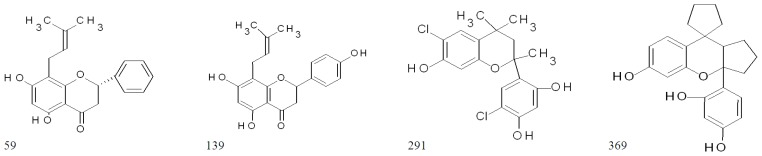
The four most active anti-biofilm flavonoids identified in this contribution.

These ten highly active flavonoids were further retested at 100 μM (Scheme 1; Table 1), and the high activity was only preserved in four molecules: two flavanones (**59**, **139**) and two flavan derivatives (**291**, **369**). Their structures are shown in Figure 4.

A literature search of flavanone glabranine **59** ((2*S*)-5,7-dihydroxy-8-(3-methylbut-2-enyl)-2- phenylchroman-4-one) promptly revealed that it is present in Mexican plants of the *Tephrosia* genus and is shown to possess inhibitory effects on the dengue virus at relatively low micromolar concentrations (25 μM) [42]. On the other hand, flavanone **139** (3,5,7-trihydroxy-2-phenylchromen-4-one), also known as the 8-prenylnaringenin, is typically found in hops and is a well-known and potent phyto-estrogen with documented *in vitro* and *in vivo* effects [43]. The side prenylation in position 8 is also present in glabranine, which suggests the importance of this side chain. The 6-prenylated naringenin (absent in this flavonoid library) has been shown to be antibacterial against suspended *S. aureus* (MIC of 18.3 μM) [44]. It has been speculated that the lipophilicity that is provided by the prenyl side chain could improve the bacterial transmembrane transport of these derivatives [45] and may tentatively facilitate the interaction of both compounds (**59** and **139**) with the biofilm matrix, in comparison to naringenin (**164**, inactive here). However, at this point, it was reasoned that compounds with other properties (such as antiviral or estrogenic) would not be selective anti-biofilms, and they may cause undesirable off-target effects. Consequently, flavanones **59** and **139** were excluded from further analysis.

Attention was then focused on the flavan derivatives, **291** (6-chloro-4-(6-chloro-7- hydroxy-2,4,4-trimethylchroman-2-yl)benzene-1,3-dioland) and **369** (4-(6-hydroxyspiro[1,2,3,3a,9apentahydrocyclopenta[ 1,2-b]chromane-9,1′-cyclopentane]-3a-yl)benzene-1,3-diol). Upon a literature search, no bioactivity data were found associated with these molecules. This search included the freely available Antimicrobial Index database (http://antibiotics.toku-e.com/), which is a very complete source of antibacterial molecules, as well as the PubChem database. One factor to keep in mind is that, in general, studies on the bioactivity of flavans appear to be more scarce in comparison to other flavonoid classes [46], and fewer screenings for their antibacterial activity have been published.

### 2.4. The Two Most Potent Flavonoids: Potencies, Efficacies and Mechanistic Insights

The two identified flavans prevented bacterial colonization and decreased the viability of existing biofilms (Table 2). They also caused a reduction in biofilm biomass, and the potency values quantified with the crystal violet assay were similar to the ones presented here (results not shown). The activity order was maintained in both exposure strategies, with **291** being the most active. Interestingly, the ratios between the concentrations needed to reduce 50% of the viable cells in the existing biofilm, and those needed to prevent 50% of biofilm formation varied between 2.7 and 3.4; that is a great advantage if compared with the antibiotic, penicillin G (Table 2).

The same viability staining was applied to measure the efficacy these flavonoids had against suspended cells, so the comparison of the antibacterial and anti-biofilm results could be somewhat easier. The two most active flavans were shown to display a potent antibacterial activity, with similar MIC and minimal bactericidal concentrations (MBC) values quantified on the suspended cells (Table 2). MIC values, in particular, corresponding to 7.3 μg/mL and 14.1 μg/mL for compounds **291** and **369**, respectively, are indicative of a significant antibacterial activity, judging from previous indications that compounds with MICs ≤ 100 μg/mL are considered noteworthy and those with MICs ≤ 10 μg/mL are regarded to be very interesting [15,47]. Compound **291** caused 50% inhibition of biofilm formation at 10 μM, and it reached more than 90% of biofilm viability inhibition with a minor concentration increase (at 13 μM), which was very close to the MBC value quantified here (15 μM). Similarly, compound **369** had a half maximal inhibitory concentration (IC_50_) of 17.7 μM and caused 90% inhibition of biofilm formation at 40 μM, corresponding to its MBC.

Based on how close the anti-biofilm IC_90_ and the MBC values were, it could be suggested that these flavans caused a significant killing upon direct interaction with the suspended cells, resulting in a reduction of the suspended viable cell density initially present to attach to the polystyrene surface of the microtiter well plates to begin the biofilm formation process. However, on the other hand, a former contribution demonstrated a very significant biofilm formation after 18 h, even if only a low concentration of viable bacterial cells (10^3^ colony forming units, CFU/mL) was present at the beginning of the incubation period [20–23]. Thus, reducing the starting population of viable suspended bacteria even by 3-log units would still not be enough to explain the ability of these compounds to significantly inhibit bacterial colonization and biofilm formation. Consequently, it is likely that in the presence of **291** and **369**, some cells are still able to attach and form biofilms, but their maturation process is significantly hampered, resulting in the presence of less viable cells in the bacterial biofilm core, after 18 h. This possibility is supported by the fact that **291** and **369** were also potent in the post-exposure assay (when they only encounter bacterial biofilms), as previously discussed at the beginning of this section.

Next, the killing efficacy was studied. Formed *S. aureus* biofilms (18 h) were exposed to various concentrations of the two flavans during 24 h, and the density of biofilm bacteria left on the wells and bacteria in the planktonic phase were measured using agar plate counts (Table 3). The log R parameter was calculated by comparing the log counts from the treated wells to untreated controls, as in [48].

This experiment intended to clarify the ability of these compounds to reduce the viable bacterial burden in the biofilm phase using the reference or golden standard method for bacterial quantification (the agar plate counts). Due to the laboriousness and low throughput of this method, it is only applicable for follow-up studies in screening campaigns for chemical libraries, at the point when active ones have been selected and a reasonable amount of samples are to be handled. Compounds causing at least a 3-log reduction are of greater interest, since a 3-log reduction of the biofilm burden is highly desirable to assist the immune system in clearing the remaining pathogens *in vivo*, in immunotolerant biofilm infections [49,50].

Furthermore, in this experiment, the remaining viable bacteria in the planktonic phase were quantified. Although planktonic cells are removed (and flavonoids are added), once the biofilms have been formed for 18 h, the continuation of the experiment for an additional 24 h results in space limitations for the existing *S. aureus* biofilms, due to the relatively small size of the wells. A fraction of the biofilm population then gets detached and can switch into the suspended state to maintain the stability of the established biofilms. Accordingly, at the end of the post-exposure time, a high concentration of viable biofilm bacteria is typically recovered from the untreated wells (4 × 10^9^ CFU/mL, corresponding to 5 × 10^8^ CFU/cm^2^), as well as of suspended bacteria from the same wells (6 × 10^9^ CFU/mL).

Flavans **291** and **369** caused more than 3-log reduction of the biofilm viable core at concentrations of 200 and 100 μM, respectively, indicative of 99.9% killing. Applying flavan **291** resulted in a similar log reduction of biofilm and planktonic cells throughout the entire concentration range, confirming that this compound has both antibacterial and anti-biofilm activity and that it can be equally effective at targeting both, under the same experimental conditions. Flavan **369**, on the other hand, was more effective in reducing the viable suspended bacteria (achieving the maximum log R values with no detectable living colonies) than the viable core of bacterial cells residing on the *S. aureus* biofilms. This suggests instead that **369** is a flavan behaving more as an antibacterial than an anti-biofilm compound.

If only the antibacterial activity is to be taken into account, **291** ranks within the top 10 most active flavonoids reported in the literature (reviewed by Cushnie and Lamb [15]). However, it is the fact that **291**, as well as **369** are also potent anti-biofilm agents that makes them highly interesting. Bacteria are switching between single-cell and biofilm states in host organisms, and the predominant lifestyle can dynamically change depending on various complex factors. Thus, compounds that can successfully act on both at fairly similar concentrations are the most promising starting point for more feasible candidates for antibacterials.

## 3. Experimental Section

### 3.1. Compounds Collection

The studied collection consisted of 500 synthetic and natural flavonoids that are commercially sold by TimTec (www.timtec.net). Structures of all compounds can be retrieved from the vendor’s database (http://www.echemstore.com/). Flavonoids with 9 different basic cores are present in the library (flavanones, flavones, chalcones, flavonols, dihydroflavonols, flavans, anthocyanins, isoflavonoids and neoflavonoids). The list of all tested compounds is presented in Table S1, as well as their trivial and short names, when available. The average molecular weight of the collection is 342.8 g/mol, and the averages of predicted log*P* and log*S* are 3.49 and −4.46, respectively, as provided by the supplier. Compound identities were confirmed by the supplier, using Nuclear Magnetic Resonance spectroscopy (NMR) (300 MHz or higher) and Liquid Chromatography-Mass Spectrometry (LC/MS). Compound purities were measured using High-Performance Liquid Chromatography (HPLC) and ensured to be over 95%. All compounds were dissolved in dry DMSO to a concentration of 20 mM and were maintained in matrix storage tubes (Thermo Fisher Scientific, Waltham, MA, USA) at −20 °C. From those stocks, the compounds were added directly into the assay plates containing the culture media or further dilutions in dry DMSO were made for the bioactivity follow-ups with the most active compounds. Additional amounts of flavans **291** (company ID: ST075672) and **369** (company ID: ST081006) were purchased from TimTec for the characterization studies. Structural validation for these two compounds is presented in Table S4.

### 3.2. Biofilm Assay

*S. aureus* (ATCC 25923 and Newman, both clinical strains of human origin) were used for the primary screening of the flavonoids collection. Bacteria were cultured in 30 g/L tryptic soy broth (TSB, Fluka Biochemika, Buchs, Switzerland) under aerobic conditions at 37 °C, 200 rpm for 4 h to reach exponential growth. Biofilms were formed according to the conditions previously described [23]. Briefly, exponentially grown bacteria (10^6^ CFU/mL, 200 μL) were added to flat-bottomed 96-well microplates (Nunclon Δ surface) (Nunc, Roskilde, Denmark), and biofilms were formed at 37 °C, 200 rpm for 18 h in 30 g/L TSB.

### 3.3. Exposure to Flavonoids

The anti-biofilm effects of the flavonoids were examined prior to biofilm and post-biofilm formation, as recently described in [23]. During the pre-exposure, compounds and bacterial suspension were added, and the effects were examined after incubation at 37 °C, 200 rpm for 18 h. In the post-exposure, biofilms were formed first during 18 h (37 °C, 200 rpm, as described earlier), flavonoids were added and plates were incubated for 24 h at 37 °C, 200 rpm. Penicillin G, used as the control antibiotic in all experiments, was prepared in Mueller-Hinton Broth (MHB) and tested at 400 μM. Untreated biofilms, cell-free samples with TSB and biofilms exposed to 2% DMSO were included as controls. Initial screening of the entire collection was conducted at a final compound concentration of 400 μM for both bacterial strains. The reconfirmation trial was performed for selected compounds at 100 μM.

### 3.4. Quantification of Biofilms

Biofilm viability and biomass were measured using resazurin and crystal violet staining assays, as previously described [23]. Biofilms were first stained with resazurin 20 μM (Sigma-Aldrich, St. Louis, MO, USA) for 20 min at room temperature (RT), (darkness, 200 rpm), followed by the measurement of the fluorescence emitted by the resorufin (the reduced form of the resazurin), at λ_exc_ = 560 nm and λ_em_ = 590 nm using a Varioskan reader (Thermo Fisher Scientific, Vantaa, Finland). The resazurin stain was then removed and replaced with crystal violet, which was added in the sample plate using a Multidrop^®^ Combi dispenser (Thermo Fisher Scientific, Vantaa, Finland) and incubated for 5 min (RT, static conditions). After staining, biofilms were washed three times with Milli-Q (MQ) water with the aid of a Biomek 3000^®^ liquid handling workstation (Beckman Coulter Inc., Fullerton, CA, USA). The remaining dye was recovered by the addition of 96% ethanol, and after 1 h, the absorbance was measured at λ = 595 nm using a Varioskan plate reader.

### 3.5. Anti-Biofilm Potency and Efficacy Testing

Anti-biofilm potencies of selected compounds (coded **291** and **369**) were measured using 18 concentration points within a concentration range of 10–400 μM in both exposure schemes. Anti-biofilm efficacies were measured using the Log Reduction (Log R) assay [48], which is based on viable cell counts in tryptic soy agar (TSA) plates. At the end of the 24 h exposure to these compounds, planktonic cells were transferred to sterile plates, while biofilms were scraped off the wells in 100 μL TSB using sterile plastic sticks and rinsed with an additional 100 μL of TSB. To disperse the bacterial aggregates, samples were immersed in a high power ultrasonic bath (Bandelin Sonorex Digitec, Zurich, Switzerland) using an in-house built-in device that allowed them to be in full contact with the water. Sonication was performed at RT (5 min, 35 kHz). The disaggregated biofilms, as well as the planktonic cells, were serially diluted, spread onto TSA plates and incubated at 37 °C overnight. The log_10_ density of viable cells (CFU/mL) in treated and control wells were determined, and Log R was calculated from the difference of the log_10_ density on the untreated biofilms (or the planktonic phase of the corresponding wells) and the log_10_ density on the biofilms treated with the flavonoids (or the planktonic phase of the corresponding wells). To confirm that all biofilms have been recovered, the scraped wells were stained with resazurin (as in Section 3.4).

### 3.6. Bacteriostatic and Bactericidal Effect on Planktonic Cells

Minimal inhibitory concentrations (MIC) of **291** and **369** for planktonic bacteria were first measured. After exposing biofilms to the flavonoids (at 37 °C for 18 h), the planktonic phase was transferred to sterile 96-microtiter well plates, and quantitative readouts were obtained (at λ = 620 nm) using the Varioskan Flash Multimode Plate Reader (2.4.3.37 software, Thermo Fisher Scientific Oy, Vantaa, Finland, 2004), as in [23]. The minimal bactericidal concentrations (MBC) were also measured. For this purpose, after making the MIC determinations, suspensions were mixed with resazurin 400 μM (10 μL), to reach a final concentration of 20 μM. Plates were then incubated in the darkness, RT, with 200 rpm shaking for approximately for 4 min, followed by the measurement of fluorescence (λ_exc_ = 570 nm; λ_em_ = 590 nm) using the Varioskan Flash Multimode Plate Reader (2.4.3.37 software, Thermo Fisher Scientific Oy, Vantaa, Finland, 2004).

### 3.7. Statistical Analysis and Data Processing

At least four replicates per treatment were included in each plate, and two separate experiments (biological replicates) were always run. Anti-biofilm activities were expressed as inhibition percentages of the untreated biofilms (Equation 1). The potencies of the anti-biofilm effects (IC_50_) of **291** and **369** were calculated from 18 concentration points via a non-linear regression analysis (sigmoidal dose-response with variable slope), and the results are presented with 95% confidence intervals. Below, μ_max_, and μ_min_ represent the means (average) of the signals (the fluorescence of the reduced resazurin or absorbance of the crystal violet stained biofilms) recorded in untreated biofilms and TSB controls, respectively.

(1) $$\text{Inhibition }\%=[(\mu_{\text{max}}-\mu_{\text{treated well}})/(\mu_{\text{max}}-\mu_{\text{min}})]\times 100\%$$

Quality control of the screening process was done via the calculation of the screening window coefficient (*Z*′) and signal-to-background (*S*/*B*) and signal-to-noise (*S*/*N*) ratios, as defined by [51,52]. Calculated parameters during the first screening behaved as follows: *Z*′ > 0.6; *S*/*B* > 16 and *S*/*N* > 7. All data processing and statistical analysis was done with Microsoft Excel 2010 software and GraphPad Prism version 5.0 for Mac, GraphPad Software (San Diego, CA, USA, 2009), respectively.

## 4. Conclusions

The body of data produced here serves three main purposes. First, it offers an improved methodological workflow for anti-biofilm phenotypic screens of chemical libraries. By using methods optimized for biofilm quantification in the context of chemical screening and by designing the study with two exposure modes and two *Staphylococcus* spp. Strains, the prior flaws of antibacterial studies of flavonoids have been minimized. Furthermore, to improve data analysis, the probable connections between the anti-biofilm and antibacterial properties of the flavonoids were investigated, which is often times ignored in the scientific literature. This is particularly relevant for the proper interpretation of the bioactivity data obtained from flavonoids, but also from other natural products with reported antibacterial effects, for instance, phenolic acids, catechins and phytosterols, among others.

Second, it provides a large anti-biofilm bioactivity dataset for flavonoids. An effort was made to highlight relevant flavonoids and to compare current results with prior contributions, so that a better understanding of their bioactivity profiles was achieved. The dataset built here will also hopefully pave the way for future structure-activity relationship (SAR) studies and structural optimizations.

Finally, this contribution identifies two new synthetic flavans (compounds **291** and **369**) as potent antimicrobials that can successfully act on biofilms and suspended *S. aureus* and represent more feasible antibacterial candidates, to be included in a new generation of pharmaceuticals. This research may also potentially spark interest for more focused studies on this flavonoid class. More data is indeed needed on their *in vivo* bioavailabilites and safety profiles, which will benefit a better exploitation of flavans as drug leads.

## Figures and Tables

**Table 1:** Results from the first screening assay and reconfirmation trial for the ten flavonoids originally classified as highly active (summarized in Scheme 1).

Code	Company ID	Class	Inhibition of biofilm formation (%)					

			Primary screening at 400 μM				Reconfirmation trial at 100 μM	

			Prior-to-exposure		Post-exposure		Prior-to-exposure	Post-exposure

			strain 1	strain 2	strain 1	strain 2	strain 1	
33	ST014848	isoflavone	93.8	93.8	94.5	94.9	19.7 ± 5.6	0
59	ST024709	flavanone	94.1	92.6	94.8	94.0	95.5 ± 0.1	92.5 ± 3.2
139	ST056204	flavanone	93.8	95.1	86.9	94.3	95.0 ± 0.1	93.6 ± 1.1
291	ST075672	flavan	93.2	92.6	94.6	94.7	95.4 ± 0.1	94.1 ± 1.2
369	ST081006	flavan	93.6	96.1	93.9	90.3	95.3 ± 0.2	93.3 ± 1.7
424	ST092293	chalcone	93.8	94.3	93.7	90.2	15.9 ± 5.1	0
432	ST093738	flavan	94.1	94.0	89.4	88.1	0	0
446	ST098360	flavanone	92.0	91.2	92.9	92.9	0	0
464	ST095411	chalcone	93.7	93.7	90.5	89.4	0	0
469	ST095417	chalcone	94.8	94.1	88.8	90.0	0	0

Strains 1 and 2 refer to *S. aureus* ATCC 25923 and Newman clinical strains, respectively.

**Table 2:** Anti-biofilm and antibacterial potencies of the top two flavonoids (flavans **291** and **369**). MIC, minimal inhibitory concentrations; MBC, minimal bactericidal concentrations.

Code	Effects on biofilms (IC_50_, μM) (μg/mL) (95% confidence intervals)		Effects on suspended bacteria	

	Prior-to-exposure	Post-exposure	MIC, μM	MBC, μM
**291**	**10.2** (3.77) (8.9–11.6)	**27.9** (10.3) (22.9–33.9)	**20** (7.38)	**15** (5.54)
**369**	**17.7** (6.24) (12.7–24.8)	**60.5** (21.3) (49.6–73.9)	**40** (14.1)	**40** (14.1)
**Penicillin G**	**0.13** (0.048) (0.12–0.14)	45.2%*	**0.12** (0.045)	**0.13** (0.048)

* Percent inhibition at 5 mM. Penicillin fails to cause more than 50% of biofilm inhibition in the post-exposure assay, as previously shown in [22].

**Table 3:** Killing efficacy of flavans **291** and **369**.

Code	Concentration (μM)	Log Reduction (biofilm phase)	Log Reduction (planktonic phase)
**291**	20	0.6	0.1
	80	1.5	0.7
	200	3.5	3.5
	400	4.6	4.7

**369**	50	1.5	1.2
	100	3.9	4.1
	250	3.1	9.0
	400	3.9	9.0

**penicillin G**	400	1.0	4.0
